# Uterine fibroids – what’s new?

**DOI:** 10.12688/f1000research.12172.1

**Published:** 2017-12-07

**Authors:** Alistair R.W. Williams

**Affiliations:** 1 Department of Pathology, Royal Infirmary of Edinburgh, University of Edinburgh, 51 Little France Crescent, Edinburgh, EH16 4SA, UK

**Keywords:** uterine fibroids, leiomyomas, molecular, life cycle, management

## Abstract

Uterine fibroids are the commonest benign tumours of women and affect all races with a cumulative lifetime risk of around 70%. Despite their high prevalence and the heavy economic burden of treatment, fibroids have received remarkably little attention compared to common female malignant tumours. This article reviews recent progress in understanding the biological nature of fibroids, their life cycle and their molecular genetic origins. Recent progress in surgical and interventional management is briefly reviewed, and medical management options, including treatment with selective progesterone receptor modulators, are also discussed.

## Introduction

Uterine fibroids (leiomyomas) are benign monoclonal tumours of smooth muscle, taking origin in the myometrium. They are the commonest benign tumours of the uterus, and are typically round well-circumscribed masses. They are usually multiple, and can range in size from a few millimetres to massive growths of 20cm diameter and more. The aetiology is largely unknown, but they are oestrogen- and progesterone-dependent tumours, very rare before menarche, common in reproductive life, and frequently regress in size after menopause ^1^.

By age 50, it is estimated that 70% of women will have one or more uterine fibroids, with around 30% of patients symptomatic and requesting treatment. Women of all races are affected, but fibroids are commoner, and develop at an earlier age, in women of African origin ^2^. By age 35 years, 60% of African-American women will have fibroids, compared to 40% in Caucasian women of the same age. Other risk factors include age (increasing incidence with age up to the menopause, then usually decreasing in size), nulliparity, genetic factors, early menarche, caffeine, alcohol, obesity and hypertension ^3^.

Symptoms of fibroids are abnormal uterine bleeding, pelvic pain, dyspareunia, obstructive effects on bladder or rectum, and infertility. Fibroid size does not necessarily determine the severity of clinical symptoms. In a large online survey conducted in eight countries with at least 2,500 participants in each country (4000 in USA), 59.8% of women with a diagnosis of uterine fibroids self-reported heavy and prolonged vaginal bleeding compared to 37.4% in those without fibroids ^4^. Pelvic pain at various times in the menstrual cycle and during sexual intercourse were also significantly increased in fibroid patients. Excessive vaginal blood loss can lead to severe anaemia which can even be life-threatening, yet some patients do not recognise the severity of the problem, may consider their blood loss to be normal, and do not seek help ^5^.

Uterine fibroids place a large economic burden both on the women who suffer from them, and on the health systems and societies in which they live. Symptoms may lead to significant loss of working time, and in a large survey 24% of women perceived symptoms as a contributing factor in failure to achieve career aspirations ^6^. Direct surgical costs alone are high – in the USA, 200,000 hysterectomies are performed annually for fibroids ^7^, and when medications, inpatient and outpatient hospital attendances are added, the annual costs are estimated at between 4–9 billion US dollars ^8^. These costs do not include lost work time, and other consequences such as spontaneous abortions, pre-term delivery and Caesarean sections.

Imaging techniques are the mainstay of diagnosis, with transabdominal or transvaginal ultrasound the most commonly used modality, as it is widely available, inexpensive and usually definitive in diagnosis. MRI may be used to delineate the number, size and location of fibroids in certain cases, and hysteroscopy may be useful to distinguish between subendometrial fibroids and large endometrial polyps. Neither imaging nor hysteroscopic methods are currently reliable in distinguishing benign fibroids from sarcomatous uterine tumours.

Surgical treatment takes the form of hysterectomy or myomectomy, the choice depending on the size, number and extent of fibroids, and on the patient’s wishes with regard to fertility. Hysteroscopic or laparoscopic myomectomy are considered safe and effective options, but laparoscopic hysterectomy is usually still the standard surgical option in women who do not wish to retain fertility ^3^. It should be noted, however, that hysterectomy is not free from short term and long term sequelae – 1 in 30 women suffers a major adverse event, and mortality may be between 0.4-1.1 per 1000 operations ^9^. Non-surgical interventional treatments also include uterine artery embolization (UAE), and high-frequency MR-guided focussed ultrasound surgery.

Until recently, medical management of fibroids was largely confined to symptomatic treatment of pain and bleeding, and the use of gonadotropin-releasing hormone (GnRH) analogues. The latter lead to a hypo-oestrogenic state, fibroids undergo shrinkage, and blood loss and anaemia can be corrected, but duration of treatment is limited by side effects of menopausal symptoms and loss of bone mineral density.

More recently, a newer group of agents, the selective progesterone receptor modulators (SPRMs), have shown considerable effectiveness in the medical management of fibroid patients ^10^. As well as their effects on fibroid shrinkage, in most patients SPRM treatment leads to rapid control of heavy menstrual bleeding, and correction of anaemia. Oestrogen levels remain at around mid-follicular levels, and, as a consequence, menopausal symptoms and bone loss are not encountered regularly.

## Histopathology

Fibroids are correctly known as leiomyomas, being benign tumours of smooth muscle, taking origin in the myometrium. As the fibroid grows, the cells differentiate into four different cell types that can be reliably characterised: smooth muscle cells, vascular smooth muscle cells and two different subpopulations of fibroblasts. It has been shown that all four cell types derive from a single clonal origin ^11^.

Macroscopically, the lesions are usually multiple, pale, firm and rubbery, with a whorled cut surface, well demarcated from adjacent myometrium. There may be areas of mucoid change, haemorrhage, or necrosis and calcification visible on gross inspection. Microscopically, they are composed of spindle cells arranged in fascicles that interweave to form a circumscribed lesion. Mitotic activity may be observed, but there are usually less than 5 mitoses per 10 high power fields (HPF), and no atypical forms. Mitotic activity is significantly higher in the secretory phase of the cycle ^12^, an observation that suggests importance of progesterone and its receptor PR in fibroid growth. There is a great degree of variability in the amount of extracellular matrix and collagen in fibroids, leading to considerable heterogeneity in histological patterns. Degenerative changes may be superimposed, including hyaline and myxoid change, hydropic degeneration, necrosis and calcification.

Notwithstanding this variability in the usual type of leiomyoma, there are several distinct histological variants that may cause some diagnostic difficulty to the histopathologist. *Cellular leiomyoma* is significantly more cellular than the usual type, but shows no nuclear atypia, a low mitotic index (4 or less mitoses per 10 HPF), and no necrosis. *Leiomyoma with bizarre nuclei* (previously termed atypical or symplastic leiomyoma) characteristically shows highly pleomorphic extremely bizarre nuclei, often in a background of more typical leiomyoma cells. Mitotic activity is usually low, but karyorrhexis may mimic atypical mitoses, and the histopathologist must be cautious not to diagnose sarcoma, as these are benign lesions. *Mitotically active leiomyoma* shows a high mitotic index (>10 mitoses per 10 HPF), but no other concerning features, with an absence of nuclear atypia and necrosis. These are likely endocrine related, as they are seen in the reproductive age group, and have been reportedly associated with hormone therapy. *Dissecting (‘cotyledenoid’) leiomyoma* is a rare variant which shows locally invasive growth sometimes extending outwith the uterus, often with a prominent degree of hydropic change. *Diffuse leiomyomatosis* is a rare condition in which multitudes of benign-appearing leiomyomatous nodules blend with uterine smooth muscle, and may extend beyond the uterus into the peritoneal cavity forming tumour-like nodules, grossly resembling disseminated gynaecological cancers. The process is benign, and surgical removal is curative.

An uncommon but troublesome group of tumours show histological appearances that may arouse concern about possible leiomyosarcoma, but which fall short of definitively malignant lesions. Described as *atypical smooth muscle neoplasms*, or smooth muscle tumours of uncertain malignant potential (STUMP), such lesions show an intermediate level of mitotic activity (5 – 10 mitoses per 10 HPF), variable necrosis or myxoid change, and a degree of nuclear atypia, sometimes with epithelioid cell morphology. The prognosis of such tumours is unpredictable, but recurrence occurs in approximately 10 – 15% of cases. A promising approach to prediction of outcome in such lesions has recently been described ^13^ in which comparative genomic hybridisation was used to clearly stratify a series of uterine STUMP into two separate prognostic groups: one with prognosis similar to leiomyoma, the other with outcome similar to low grade leiomyosarcoma.

Leiomyosarcoma is a frankly malignant neoplasm of smooth muscle origin. Whilst most appear to occur *de novo* from myometrium, there is evidence that up to 20 – 30% may arise from pre-existing benign smooth muscle tumours (see below). This must be a rare event, considering how common benign fibroids are and the rarity of leiomyosarcoma.

## Life Cycle of Fibroids

A careful morphological review ^14^ led to the hypothesis that fibroid formation may represent an abnormal response to injury. This proposes that normal myometrium may be subject to repeated injury through vasoconstriction and hypoxia during menstruation, and that development of fibroids may represent a reaction to that injury. There are intriguing parallels with processes of wound healing, keloid formation and even the reaction to injury occurring in blood vessels in the formation of atherosclerosis. Additional evidence from Ciarmela’s group ^15^ suggests the action of an inflammatory trigger to excessive production of extracellular matrix by activated myofibroblastic cells in fibroids.

Uterine fibroids have a self-limited life cycle of proliferative growth, synthesis of collagen, increasing deposition of extracellular matrix, decreasing vascularity, and ultimately senescence and involution through ischaemic degeneration and inanition ^14^. Four phases in the life cycle of fibroids have been described, defined somewhat arbitrarily and representing a continuous process, progressing through phenotypic transformation of the proliferating contractile myocyte and evolutionary selection of a single clone. There is increasing deposition of collagen, and as the process of fibroid growth and development evolves, the phenotype of the clonally proliferating myocytes changes from contractile to collagen synthesising, with significant elaboration of extracellular ground substance. Myocytes become separated from vessels by increased amounts of extracellular matrix, and angiogenesis does not keep up with the increasing size of the fibroid. Ischaemia eventually occurs, and there is cessation of myocyte proliferation and cellular atrophy. In the end stage, there is abundant hyaline matrix enclosing islands of atrophic myocytes, and there may be necrosis and calcification. Processes of cell death, resorption and reclamation now occur, termed ‘inanosis’ by the authors. These differ from necrosis and apoptosis in their morphology, in their long, protracted durations, and in the absence of any inflammatory or phagocytic response to cell death.

## Genetic and Molecular Aspects of Aetiology

Several lines of evidence point to a significant genetic predisposition to development of uterine fibroids. Women with first degree relatives having fibroids have an increased incidence ^16^, and monozygotic twins have higher concordance for fibroids than dizygotic ^17^. Up to around 50% of uterine leiomyomas show cytogenetic alterations, including trisomy of chromosome 12, deletions in the long arm of chromosome 7, rearrangements of 12q15 and mutations in *MED12* and *HMGA2* genes. There is evidence for the existence of a population of cells with stem or progenitor cell characteristics, which can be isolated from normal myometrium and from leiomyoma tissues ^18^. It is hypothesised that activating mutations occurring in this cell type give rise to the clonal population of myocytes making up the leiomyoma. Intriguingly, a recent study using whole genome sequencing of uterine leiomyomas showed that multiple fibroid nodules within the uterus can be clonally related, indicating a single cell origin of multiple leiomyomas ^19^. This study also reported the occurrence in fibroids of complex chromosomal rearrangements resembling chromothripsis, apparently occurring as a single chromosomal shattering event with up to 20 or more double-stranded breaks, followed by random reassembly. The authors suggest that tumour formation occurs when reassembly leads to the juxtaposition and activation of tumour-promoting genes.

Epigenetic mechanisms are also likely to have a key role in fibroid formation. Several tumour suppressor genes have been shown to be abnormally hypermethylated in fibroids compared to adjacent myometrium ^20^, as are collagen-related genes and a subset of ER response genes ^21^.

### *MED12* 

Exome sequencing a small series of leiomyoma tissues identified a high frequency of somatic mutations in *MED12* (also known as mediator complex subunit 12) gene ^22^, and a subsequent larger survey of 225 fibroids from 80 patients identified *MED12* mutations in 70% ^23^, making *MED12* the most frequently altered gene in leiomyomas. *MED12* is an X-linked gene that encodes a subunit of the mediator complex that is central to regulation of transcription, and is a crucial element in canonical *WNT* signalling, known to interact with β-catenin. Most mutations occur in a highly conserved area of exon 2 of the gene, with around 50% occurring as mis-sense mutations of codon 44, and it has been suggested that these may represent ‘gain of function’ alleles ^24^. Less frequently, mutations occur at the intron 1/exon 2 boundary, and even more rarely in exon 1, respectively. There is an inverse correlation between presence of *MED12* mutation and leiomyoma size, suggesting that lesions of differing sizes may have different aetiological pathways. *MED12* mutations seem relatively specific for leiomyoma, and also occur in around 10 – 20% of leiomyosarcomas ^25, 26^. However, the same type of mutation has also been found in chronic lymphocytic leukaemia ^27^, and malignant phyllodes tumour of the breast ^28^.

### *HMGA2* 

Cytogenetically visible alterations in 12q14-15 and 6p21 have been observed in leiomyomas, and rearrangements at these loci map to genes encoding high mobility group proteins *HMGA2* and *HMGA1,* respectively, leading to their overexpression. Overexpression of HMGA2 was found to be the second most frequent genetic alteration in leiomyomas, being present in 7.5–10% ^26^. Overexpression was exclusively found in leiomyomas that did not have mutation of *MED12*, indicating the likelihood of two separate and mutually exclusive pathways of fibroid development ^29^. Each group has differing global gene expression profiles, and further evidence indicates that leiomyomas with alterations of *MED12* and *HMGA2* show different behaviours. In a study of 289 fibroids from 120 patients, it was found that over 85% of *MED12* mutated lesions occurred as multiple uterine nodules, whereas 70% of *HMGA2* mutated lesions were single nodules ^30^.

Rarely, uterine leiomyomas may be part of the hereditary leiomyomatosis and renal cell cancer syndrome caused by heterozygous germline mutations in the *fumarate hydratase* ( *FH*) gene. The disorder has an autosomal dominant pattern of inheritance, and is clinically characterised by the occurrence of multiple (10 to over 100) cutaneous leiomyomas, often painful, occurring in a segmental pattern on trunk and extremities. Leiomyomas in this syndrome present a unique global gene expression profile, without overlap with those associated with *MED12* or *HMGA2* mutations ^23^.

## Advances in surgical and interventional management of fibroids

For many years, hysterectomy has been the treatment of choice for uterine fibroids, and is still the most commonly used treatment. Laparoscopic hysterectomy rates may exceed 90% in some departments, but other surgical and interventional treatments are increasingly available ^3^. The treatment selected often depends on the patient’s age, her willingness to undergo what is perceived to be a major surgical procedure, and her desires for future fertility or complete amenorrhoea. Guidelines exist in the literature, but there are few clinical trials comparing different treatments.

Hysteroscopic myomectomy is suitable for fibroids of certain sizes and locations, being most suited to smaller pedunculated submucous lesions which can be removed by transection of the base with a resectoscopic loop. Some smaller intramural fibroids may be removed hysteroscopically in one- or two-step procedures that involve slicing the lesion into chips, or use of an intrauterine morcellator.

Laparoscopic myomectomy is technically more challenging than open laparotomy, but reproductive outcomes are similar, post-operative morbidity is much less and recovery times much shorter. Fibroids are usually removed with the aid of a power morcellator. Morcellation has the drawback of potential peritoneal dissemination of unrecognised uterine sarcoma, and although the risk may have been overemphasised, it remains a theoretical concern. While recognising the difficulty of histopathological diagnosis in specimens obtained after power morcellation, a large series of 10,731 laparoscopic hysterectomies ^31^ found the incidence of malignancy in morcellated surgical specimens to be 0.06% (six cases).

Non-surgical interventional treatments include UAE, an effective and safe alternative to hysterectomy in women in whom retention of fertility is not a priority, although there is little evidence of any poorer fertility outcome with UAE compared to myomectomy ^32^. Although this treatment has similar outcomes to surgery in terms of patient satisfaction, risk of major complications and fertility outcome, there is a higher rate of minor complications and subsequent surgical intervention within two to five years, with between 15 and 32% of women requiring further surgery ^33^.

High frequency magnetic resonance guided focussed ultrasound is a technique whereby ultrasonic energy is directed with MR guidance to within the fibroid, where thermal ablation by coagulative tissue necrosis occurs. The method is not yet widely used, as it is currently expensive, is suitable for only a minority of fibroid patients, and has unknown implications for future fertility ^34^.

## Medical management of fibroids

First line management of uterine fibroids usually involves symptomatic treatment of heavy menstrual bleeding, with use of inexpensive non-steroidal anti-inflammatory drugs (NSAIDs), antifibrinolytic agents including tranexamic acid, or contraceptive steroids including the levonorgestrel intrauterine system (Mirena), the latter only suitable for patients in whom the uterine cavity is not distorted by fibroids ^35^. Although bleeding symptoms may be alleviated, there is no evidence of fibroid shrinkage, indeed there is reason to believe that progestogen therapy may induce proliferation of leiomyoma cells. Shrinkage of fibroids can, however, be achieved by treatment with GnRH agonists, or with SPRMs.

Continuous administration of a GnRH agonist leads to downregulation of pituitary GnRH receptors, with consequent decreased production of FSH and LH, and subsequently of ovarian steroids. Treatment for three to six months has been shown to result in decreased uterine and fibroid size, and duration of hospital stay after surgery ^36^. However, the hypo-oestrogenic state induced by GnRH agonist treatment results in menopausal side effects, including loss of bone mineral density, that limit treatment duration usually to six months or less. While there is some evidence that add-back therapy can offer some advantages in these situations ^37^, unwanted side effects of therapy with GnRH analogues remain a problem. It has also been observed that rapid regrowth of fibroids occurs after cessation of GnRH treatment.

### Selective progesterone receptor modulators in management

The advent of SPRMs has opened a new and promising avenue of treatment for many patients. The effectiveness of these agents is based on the premise that fibroids show progesterone dependence, and blockade or modulation of progesterone activity at PR results in cessation of proliferation and induction of apoptosis in the fibroid, with consequent shrinkage. SPRMs also rapidly induce amenorrhoea in most patients, providing additional welcome symptomatic relief of bleeding. The mechanism whereby amenorrhoea is induced remains unknown, but it is believed to be a direct effect on the endometrium. Clinical trials of SPRMs in treatment of fibroids have been carried out using a variety of agents, including mifepristone ^38^, telapristone acetate ^39^, asoprisnil ^40^, ulipristal acetate ^41^, and vilaprisan ^42^.

SPRMs induce characteristic morphological changes in endometrium that have not been observed with other pharmaceutical agents, and have been designated PAEC (progesterone receptor modulator-associated endometrial changes ^43^). Uninterrupted treatment with SPRMs for six months or more induces endometrial thickening, and to avoid associated complications, successful clinical trials have utilised an interrupted regime of three months on treatment followed by one month off, with menstrual shedding of the endometrium.

In 2012, following large Phase III clinical trials, ulipristal acetate was the first SPRM to be granted a licence from the European Medicines Agency for use in the pre-surgical treatment of fibroids, and it is now being used in many countries worldwide. Early Phase III trials showed that one course of 5 mg ulipristal acetate orally for 12 weeks led to a mean 20–35% reduction in fibroid volume, and that the reduction in volume was maintained for up to 6 months following end of treatment ^10, 41^. Treatment was also associated with rapid control of uterine bleeding in over 80% of patients. Subsequent trials showed that further fibroid shrinkage occurred with repeated courses, with median reduction in fibroid volume of 71.8% after 4 courses ^44^. Histopathological assessment of fibroids resected after ulipristal acetate treatment has shown induction of apoptosis and remodelling of extracellular matrix in the lesions ^45^. Subsequent studies established endometrial safety of up to eight courses, using a repeated interrupted regime of three months treatment followed by one month off treatment with menstrual shedding ^46^. As oestrogen levels are not suppressed on treatment, menopausal symptoms and bone mineral loss are not significant clinical issues. Ulipristal acetate is now licensed for repeated 12 week courses, but must be prescribed with a one month break between courses, to avoid adverse endometrial effects. A retrospective analysis of 21 patients who enrolled in two of the clinical trials of ulipristal acetate, who had myomectomies and wished pregnancy after treatment, reported successful pregnancies in 15 patients (71%), with birth of 13 healthy babies and 6 early miscarriages ^47^.

## What Does the Future Hold?

After many years of relative neglect, the pathogenesis of uterine fibroids is now receiving more attention, and we are beginning to gain a foothold in understanding the molecular genesis of these very common and troublesome tumours. These are the first necessary steps in the journey towards effective non-surgical treatment and perhaps even prevention. Excellent progress has been made in the laparoscopic surgical treatment of fibroids and this will continue, perhaps with robotic and other developments. However, the long-term goal must be to develop effective medical treatments, and the advent of SPRMs opens up the prospect of safe therapy without the troublesome side effects of previous medical treatments, with the potential to greatly improve the quality of life of huge numbers of women around the world.
